# Configurational paths to the green transformation of Chinese manufacturing enterprises: a TOE framework based on the fsQCA and NCA approaches

**DOI:** 10.1038/s41598-023-46454-9

**Published:** 2023-11-06

**Authors:** Zeyan Miao, Guohao Zhao

**Affiliations:** School of Business Administration, Shanxi University of Finance and Economics, Taiyuan, 030006 China

**Keywords:** Environmental impact, Environmental economics, Sustainability

## Abstract

In the current complex and ever-changing environment, the high-quality development of manufacturing enterprises has a long way to go. The theoretical framework based on technology, the organization and the environment (TOE) from a configuration perspective provides a new integrated theoretical perspective for studying the green transformation path of Chinese manufacturing enterprises. The research purpose of this article is to use fuzzy set qualitative comparative analysis (fsQCA) and necessary condition analysis (NCA) to explore the configuration effects of various antecedents in the TOE theoretical framework of Chinese manufacturing enterprises, “technology (green technological innovation, digital transformation)-the organization (redundant resources, supply chain concentration, information transparency)-the environment (environmental governance pressure)”, to achieve a green transformation. The research findings show that no single factor is a necessary condition for achieving a high level of green transformation. There are six ways to achieve high green transformation levels for manufacturing enterprises, namely, technology-information collaborative green transformation, technology-supply chain collaborative-driven green transformation, digital-organization collaborative-driven green transformation, innovation-organization collaborative-driven green transformation, organization-environment collaborative-driven green transformation, and full-level multifactor collaborative-driven green transformation. The research conclusion will further expand research on the green transformation of enterprises and provide useful and practical references for green transformation paths of enterprises.

## Introduction

At the 2020 United Nations General Assembly, the Chinese president proposed the goal of achieving a carbon peak by 2030 and carbon neutrality by 2060 (“dual carbon” goal). The 2023 *Chinese Government Work Report* further emphasizes the need to promote energy conservation and carbon reduction in key areas, continue to fight the battle for protecting blue skies, clear waters, and clean land, and promote the green transformation of the entire society. Driven by the dual carbon goal and to promote the green transformation of the entire society, manufacturing enterprises are exploring how to transform to form a green and low-carbon core competitive advantage, enhance enterprise value, and achieve sustainable development. From a practical perspective, the extensive development model of traditional manufacturing has led to a high proportion of low-end manufacturing in the entire manufacturing industry, with enormous energy consumption intensity and intensified emissions of carbon dioxide and other exhaust gases, resulting in China's manufacturing industry being “large but not strong”^1^. Since the introduction of the new concept of “green” development in China, the government has increased its supervision of environmental pollution, and it has taken a series of environmental protection measures, effectively suppressing energy consumption and waste in the manufacturing industry, as well as greenhouse gas emissions. However, China's manufacturing industry has not yet brought to an end the situation of high energy consumption, high emissions, and high pollution from the root cause. Thus, the green transformation of Chinese manufacturing enterprises is facing a series of constraints. In addition, with the continuous impact of product iteration and the digital technology revolution, the demand for green transformation in the manufacturing industry is becoming increasingly urgent. As important implementers and micro foundations of green production, manufacturing enterprises urgently need to optimize their production processes, upgrade their industrial structures, implement clean production, and optimize environmental governance through digital transformation, green technological innovation, and other means. The green transformation and development of manufacturing enterprises are inevitably a key link and core element in achieving the high-quality development of China's social and economic development.

The green transformation of enterprises is influenced by various internal and external factors, such as green technological innovation^2^, organizational management, the supply chain^3^ and environmental uncertainty^4^. Most existing research has explored and analyzed the linear or nonlinear relationship between the antecedent conditions of technological factors, organizational factors, environmental factors and the green transformation of enterprises. These studies provide a solid theoretical foundation for this article to delve deeper into the specific green transformation paths of manufacturing enterprises. However, only exploring and analyzing the linear or nonlinear relationship between these antecedent factors and the green transformation of enterprises cannot truly clarify the mechanism through which multiple antecedent factors connect and drive this transformation. Regarding research on the green transformation of manufacturing enterprises, most scholars equate enterprise green technological innovation with enterprise green transformation and choose to use methods such as system dynamics^5^ or case studies for research^6^. In fact, enterprise green technological innovation is only one part of enterprise green transformation. The green transformation of enterprises is a green development model driven by green innovation, taking into account the financial performance, environmental performance, and value cocreation of enterprises, ultimately achieving ecological environment improvement and high-quality socioeconomic development^1^. This article still regards green technological innovation in enterprises as an important technological factor, and it takes into account the practical factor of digital transformation. It explores the paths through which these two technological factors interact with other antecedents to drive green transformation in manufacturing enterprises.

The green transformation of manufacturing enterprises is a collaborative process of multiple antecedent factors at the three levels of technology, the organization, and the environment. The framework of technology-organization-environment (TOE) theory was proposed by scholars Tomatzky and Fleischer in 1990 in their book The Process of Technological Innovation, with the aim of exploring the influencing factors of implementing enterprise technological innovation^7^. TOE theory has a good effect in explaining the causes of complex phenomena in enterprises and extracting influencing factors. In addition, this study provides a new integrated research perspective to explore in depth the antecedents and paths of green transformation in manufacturing enterprises^8^. Under the framework of TOE theory, the various factors driving the green transformation of manufacturing enterprises have complex interactions, collaborative symbiosis, and other characteristics. Research on “what combination of factors can improve the level of green transformation of manufacturing enterprises” belongs to a complex causal relationship problem, while configuration thinking focuses on “multiple causes and one result”, which helps to explain multiple concurrent causal complexity problems. The mixed use of fsQCA (fuzzy set qualitative comparative analysis) and NCA (necessary condition analysis) has been widely carried out to study the results of multiple antecedent factors working together^6^. It is an ideal method for analyzing the linkage of various factors, such as technology, the organization, and the environment, on the green transformation of manufacturing enterprises.

On this basis, this article uses the fsQCA and NCA methods to explore the complex paths of six antecedent factors driving the green transformation of manufacturing enterprises under the TOE theoretical framework, namely, “technology (green technological innovation, digital transformation)-the organization (redundant resources, supply chain concentration, information transparency)-the environment (environmental governance pressure)”. Individual antecedents cannot constitute a necessary condition for driving manufacturing enterprises to achieve a high level of green transformation. In addition, technological factors are crucial for the green transformation of manufacturing enterprises, and their role will only be weakened when environmental governance pressure is high. This study expands the perspective of research on the causes and paths of green transformation in manufacturing enterprises and provides practical guidance for the green transformation paths of manufacturing enterprises, which is conducive to enhancing the core competitiveness of such enterprises. At the same time, it also provides policy implications for the government to formulate policies for enterprise digitalization and green transformation and to cultivate the concept of enterprise digitalization and green transformation.

### Theoretical basis

#### The antecedent factors affecting the green transformation of manufacturing enterprises at the technological level

Schumpeter's “innovation” theory believes that the essence of digital transformation lies in the combination of a large number of “data” elements and other resource elements, which is an innovation that can promote the optimization of production factor allocation in production manufacturing and pollution control processes. Digital transformation is one of the technological factors affecting the green transformation of manufacturing enterprises. It is defined as the integration of digital technology in enterprise business processes^9^, thus promoting the transformation of green production and governance models and achieving the high-quality green transformation of enterprises. On the one hand, manufacturing enterprises are vigorously deploying digital technology, and the scale effect optimizes and improves the efficiency of enterprise resource allocation. On the other hand, by integrating digital technology in businesses, technological effects can improve enterprise resource and energy utilization, reduce pollutant emissions, and reduce carbon emissions. The breadth and depth of digital transformation complement each other, helping to achieve the green transformation of manufacturing enterprises^10^.

The “proactive behavior theory” of green transformation holds that the application of digital technology provides technical support for enterprises to acquire cutting-edge technologies and creates conditions for technology exchange and sharing^49^. It accelerates the implementation of the achievements of green technological innovation in both independent and cooperative innovation. Green technological innovation leads to the upgrading of manufacturing equipment technology, and enterprises fully utilize intelligent equipment to promote energy conservation and emission reduction, reduce environmental pollution, and facilitate their green transformation. The “passive behavior theory” of green transformation believes that achieving China's dual carbon goals and government environmental regulations will force manufacturing enterprises to undergo green transformation, and green technological innovation is an important engine for promoting the green transformation of enterprises. Many green and low-carbon development goals will also force enterprises to organically combine digital technology with green development, activating the potential of data elements^11^.

#### The antecedent factors affecting the green transformation of manufacturing enterprises at the organizational level

The theory of resource allocation holds that the key to achieving sustained competitive advantage for enterprises lies in how to effectively allocate and sustainably utilize the redundant or core resources of enterprises. Redundant resources are defined as the minimum resources owned by an enterprise that exceed its own survival requirements^12^. Within an enterprise, redundant resources can serve as a buffer and enhance the flexibility of the enterprise. Redundant resources can alleviate the increasing cost pressure of manufacturing enterprises in environmental improvement due to increased environmental investment, which is conducive to increasing their resource investment in green technological innovation, effectively buffering the additional cost increase caused by independent green technological innovation and the introduction of green technological innovation in the green transformation process, as well as the pressure of resource shortage in the green transformation process of manufacturing enterprises^13^. Furthermore, the existence of redundant resources will have a positive impact on the development of green product innovation, making it easier for manufacturing enterprises to achieve a green transformation^14^.

The theory of competitive advantage holds that the bargaining power of suppliers is one of the important reasons why manufacturing enterprises can obtain a competitive advantage. The core of supply chain relationship management is the integration of the supply chain, and the main result of supply chain integration is the concentration of the supply chain^15^. The smaller the value is, the lower the concentration of an enterprise's supply chain. At this point, the bargaining power of suppliers is limited, which is beneficial for reducing enterprise costs and increasing profits. Only then will enterprises have more funds to purchase intelligent devices, promote energy conservation and emission reduction in manufacturing, reduce environmental pollution, and achieve a green transformation^16^. Therefore, supply chain concentration is a major antecedent factor affecting the green transformation of manufacturing enterprises.

Traditional decision-making theory points out that decision-makers are characterized by 'bounded rationality' rather than being 'completely rational'^17^. Decision-makers cannot obtain all alternative solutions and decision-related information before making a decision. Digitalization can effectively alleviate this information asymmetry situation. In a highly complex and uncertain environment, the green transformation of enterprises requires correct decision-making in areas such as green technological innovation, green production, green management, and green supply chains. On the one hand, the digital transformation of manufacturing enterprises can obtain and collect more dynamic information and data on energy conservation, emission reduction, and environmental governance, improving the coherence and transparency of green management decisions^18^. On the other hand, digital transformation enables the analysis methods and technologies of artificial intelligence to propose intelligent green transformation plans based on dynamic information on overall energy conservation, emission reduction, and environmental governance and to monitor and quantify the decision-making results of energy conservation, emission reduction, and environmental governance in real time^19^.

#### The antecedent factors affecting the green transformation of manufacturing enterprises at the environmental level

Rational behavior theory emphasizes that the strategic decision-making of manufacturing enterprises for green transformation is a recognition of external green pressure^20^. Environmental governance pressure refers to the government formulating environmental policies and technical standards, transferring the responsibility for environmental governance and green and low-carbon development to manufacturing enterprises, and enabling them to assume environmental protection responsibilities through the supervision and management of their behavior^50^. When the government formulates stricter environmental policies and technical standards, manufacturing enterprises face significant environmental governance pressure. To achieve the legitimacy and gain the high economic benefits bestowed by the government and society, manufacturing enterprises tend to use digital technology for green transformation, that is, strong environmental protection behavior. This is also a positive response behavior of manufacturing enterprises to environmental governance pressure^21^. When the government formulates relatively loose environmental policies and technical standards, manufacturing enterprises face less environmental governance pressure. In addition, the initial digital layout of enterprises will lead to increased costs and the restructuring of the internal management order. Moreover, due to their inherent external characteristics, manufacturing enterprises will engage in speculative behavior to avoid green transformation, weakening the impact of manufacturing digitization on enterprise green transformation^22^.

## Methods

The mixed use of fsQCA and NCA has been widely used in the study of the results of multiple antecedent factors working together. FsQCA is a case-oriented research method^23^. It is based on the ideas of set theory and configuration thinking, effectively linking quantitative and qualitative research and aiming to explore the relationship between a single antecedent condition element, the combination of antecedent condition elements, and the results from a set perspective and to explain the complex causal relationship underlying the phenomenon. NCA was proposed by Professor Jan Dul from the Rotterdam School of Management in the Netherlands in 2016^24^. A necessary condition refers to the antecedent conditions and elements required to achieve a certain result. If there is no such antecedent condition, then this specific result cannot be achieved. NCA mainly analyzes the effect magnitude and bottleneck level of antecedent factors, and it can intuitively display the level of antecedent factors necessary to achieve a specific result. Introducing the NCA method into fsQCA is beneficial for improving the adequacy analysis of this study.

Here are some previous studies on the sustainable development of enterprises that used the fsQCA and NCA methods. Bao Yuan et al. explored the configuration effects of the green transformation of heavily polluting enterprises under six antecedent conditions: digital technology, R&D capability, dynamic capability, the enterprise scale, resource constraints and competitive pressures^25^. X Fan et al. used the fsQCA and NCA methods to analyze the driving effect of digital transformation on the improvement in the sustainable innovation capabilities of manufacturing enterprises^26^. In addition, the newly developed fsQCA and NCA have been applied to research in areas such as urban green development^27^ and enterprise innovation^28^.

**Figure 1:**
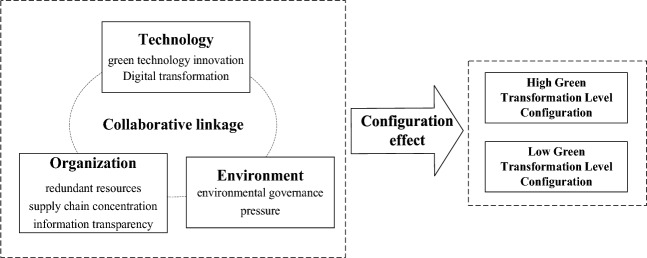
Theoretical model.

Through analysis, we found that the six antecedent factors mentioned in the second part of this article will all have an impact on the green transformation of manufacturing enterprises. Therefore, it is necessary to explore whether the six antecedent factors are necessary conditions for manufacturing enterprises to achieve a high level of green transformation. Furthermore, the configuration effect suggests that the green transformation and upgrading of manufacturing enterprises are a collaborative process of multiple antecedent factors at three levels: technological, organizational, and environmental factors. Therefore, it is necessary to explore the complex paths that drive the green transformation of manufacturing enterprises from a configuration perspective. Based on the analysis above, our research model can be obtained, as shown in Fig. 1.

### Data and variables

#### Data sources and measures

This paper selects Chinese manufacturing A-share listed companies as the research sample. Considering that COVID-19 broke out in a special period of time from 2019 to 2021 and manufacturing enterprises' operations were hit hard, to ensure the stability of the research conclusions, this paper sets the study period as 2018. This article excludes ST and * ST enterprise samples and deleted enterprises with missing data. Ultimately, it obtained 112 manufacturing enterprises as the final research case.

#### Outcome variable

The outcome variable of this article is the level of green transformation of enterprises. This article refers to the research of Wei et al. to measure the level of green transformation of enterprises from the input‒output perspective^29^. The specific operation involves using the DEA method and drawing on existing research on the green transformation of enterprises, with resource consumption, capital intensity, R&D funds, R&D personnel, and environmental protection investment as inputs. The expected output is the total industrial output value, the number of green patents authorized, and return on assets (ROA), while the unexpected output is wastewater, exhaust gas, and solid waste. The calculation is conducted using the super-efficiency SBM model. Resource consumption and unexpected output are manually organized through corporate social responsibility reports, corporate environmental reports, and corporate annual reports. The specific resource consumption is based on the research of Kumar and Jain, selecting the sum of three energy consumption indicators, i.e., industrial coal consumption, raw coal consumption, and fuel oil consumption, and converting them into standard coal (tons)^30,31^. In addition, other input and expected output data are from the CSMAR database and the website of the China National Intellectual Property Administration.

#### Independent variables

The antecedent variables at the technological level are green technological innovation and digital transformation. Regarding green technological innovation indicators, Chen et al. believe that compared to the number of green patent applications, which only shows the willingness of enterprises to engage in green innovation, the number of green patents authorized can more accurately reflect the strength of enterprises' green technological innovation^31^. Therefore, this article chooses to use the number of enterprise green patents authorized to measure green technological innovation. The number of enterprise green patents authorized comes from the website of the China National Intellectual Property Administration. The digital transformation indicators draw on the text analysis research of the highly recognized study by Li et al. and use Python software to select two subindicators based on the total frequency of keywords related to digitization in the annual reports of listed companies to characterize the level of enterprise digital transformation, as well as the breadth and depth of digital transformation. The data are sourced from the annual reports of enterprises^32^.

The antecedent variables at the organizational level are redundant resources, supply chain concentration, and information transparency. Redundant resources are measured using the mean of three dimensions, nonsedimented redundant resources, sedimented redundant resources, and potential redundant resources, following Berkes et al.^33^. The data are sourced from the WIND database. The supply chain concentration index follows the approach of Patatoukas and measures the supply chain concentration using the mean of the top 5 suppliers' and top 5 customers' Herfindahl indices. This indicator is a positive indicator, and the larger the value is, the higher the concentration of the enterprise's supply chain^34^. The data are sourced from the CSMAR database. The indicator of information transparency, based on the research of Gu et al., is the average percentage of earnings quality, information disclosure, the number of analysts tracking, the accuracy of analyst earnings prediction, and whether the auditor is one of the four major international auditors^35^. The data are sourced from the CSMAR database.

The antecedent variable at the environmental level is environmental governance pressure. This article refers to the research of Xu and uses the ratio of government environmental protection expenditure to total fiscal expenditure multiplied by 10 to measure environmental governance pressure^36^. The data are sourced from the official website of the Ministry of Finance of the People’s Republic of China.

#### Variable calibration

Before conducting fsQCA, it is necessary to convert the variables into set concepts, that is, to convert the variables into fuzzy set variables of 0–1. This article adopts the direct calibration method based on the research of Du and Kim and Ragin and Fiss to ensure the objective accuracy of the calibration process^37,38^. Considering that the variables selected in this article are relatively concentrated, the 75%, 50%, and 25% quantiles of each variable are used as anchor points and are set as fully subordinate, intersection, and completely nonsubordinate, respectively. The specific calibration of each variable is shown in Table 1.

**Table 1:** Variable calibration.

Set	Fuzzy set calibrations		
	Fully out	Crossover	Fully in
Green transformation (GT)	0.037	0.167	1.000
Green technological innovation (GI)	0.000	1.000	6.500
Digital transformation (DT)	18.000	26.000	45.250
Redundant resources (RR)	0.591	0.758	1.159
Supply chain concentration (SCC)	15.768	23.940	35.453
Information transparency (ES)	0.389	0.522	0.702
Environmental governance pressure (REGP)	0.019	0.051	0.053

## Results and discussion

### Necessary condition analysis

Before conducting research on the complex path of green transformation driven by multiple factors in manufacturing enterprises under the TOE theoretical framework, it is necessary to test whether a single antecedent variable is a necessary condition for achieving a high level of green transformation in enterprises. In fsQCA, it is currently recognized in the academic community that when the consistency is greater than 0.9, a single antecedent variable is a necessary condition for achieving the results. This article conducted a necessity test on the individual antecedents of achieving a high level of green transformation and a nonhigh level of green transformation, and the results are shown in Table 2. The necessity test results show that the consistency of a single antecedent variable as a necessary condition for achieving a high level of green transformation in enterprises is less than 0.9, indicating that the six antecedent factors, i.e., “technology (green technological innovation, digital transformation)-the organization (redundant resources, supply chain concentration, information transparency)-the environment (environmental governance pressure)”, are not necessary conditions for the outcome variable, i.e., the green transformation of manufacturing enterprises.

**Table 2:** The necessity test of a single condition for a high level of green transformation in fsQCA.

Sets of conditions	High level of green transformation (GT)		Nonhigh level of green transformation (~ GT)	
	Consistency	Coverage	Consistency	Coverage
GI	0.5783	0.5839	0.4612	0.5593
~ GI	0.5635	0.4654	0.6569	0.6517
DT	0.5682	0.5289	0.5092	0.5693
~ DT	0.5373	0.4768	0.5787	0.6168
RR	0.5713	0.5291	0.5260	0.5852
~ RR	0.5521	0.4923	0.5767	0.6177
SCC	0.5431	0.4873	0.5848	0.6303
~ SCC	0.5880	0.5411	0.5244	0.5796
ES	0.6173	0.5551	0.5268	0.5691
~ ES	0.5208	0.4782	0.5882	0.6487
REGP	0.6528	0.4734	0.7447	0.6487
~ REGP	0.5156	0.6270	0.3955	0.5778

~ means the absence of.

Furthermore, the NCA method was used for necessity testing and bottleneck-level analysis. Table 3 shows that in NCA, both the CR upper limit regression estimation method and the CE upper limit envelope analysis estimation method show that the antecedent variable d of a manufacturing enterprise's green transformation level is less than 0.1, and no antecedent condition satisfies the requirements of both effect quantity and significance. That is, there is no necessary condition for the outcome variable, i.e., the level of green transformation of manufacturing enterprises, which is consistent with the fsQCA results above. The bottleneck level refers to the minimum level value needed for the antecedent variable to reach a certain level of the outcome variable^37^.

**Table 3:** Results of necessary condition analysis.

Fuzzy-set condition	Method	Accuracy (%)	Ceiling zone	Effect size (*d*)	*P* value
GI	CR	100	0.000	0.000	0.629
	CE	100	0.000	0.000	0.599
DT	CR	100	0.005	0.005	0.270
	CE	100	0.009	0.010	0.270
RR	CR	99.1	0.004	0.004	0.329
	CE	100	0.006	0.006	0.335
SCC	CR	100	0.000	0.000	1.000
	CE	100	0.000	0.000	1.000
ES	CR	98.2	0.011	0.012	0.087
	CE	100	0.018	0.019	0.093
REGP	CR	100	0.005	0.005	0.569
	CE	100	0.009	0.010	0.569

(1) The condition variables in the table use the calibrated fuzzy membership values; (2) 0.0 ≤ d ≤ 0.1 indicates a low level; (3) the number of resamples for the permutation test when calculating the p value is 10,000.

In addition, this article uses the CR estimation method for bottleneck-level analysis. Table 4 shows that when the green transformation level of manufacturing enterprises is below 90%, no antecedent variables are necessary conditions. To achieve the 100% green transformation level of manufacturing enterprises, a 2% level of green technological innovation, a 47.5% level of digital transformation, a 26.5% level of redundant resources, an 89.6% level of information transparency, a 48.5% level of environmental governance pressure, and no bottleneck level in supply chain concentration are needed.

**Table 4:** Results of bottleneck-level analysis under the NCA method.

GT	GI	DT	RR	SCC	ES	REGP
0	NN	NN	NN	NN	NN	NN
10	NN	NN	NN	NN	NN	NN
20	NN	NN	NN	NN	NN	NN
30	NN	NN	NN	NN	NN	NN
40	NN	NN	NN	NN	NN	NN
50	NN	NN	NN	NN	NN	NN
60	NN	NN	NN	NN	NN	NN
70	NN	NN	NN	NN	NN	NN
80	NN	NN	NN	NN	NN	NN
90	NN	NN	NN	NN	NN	NN
100	2.0	47.5	26.5	NN	89.6	48.5

NN indicates unnecessary; the result was calculated using the CR method.

### Configuration analysis

**Figure 2:**
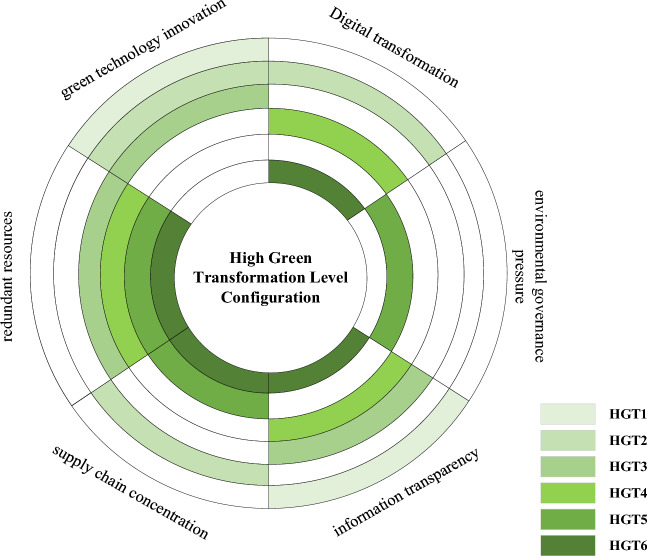
Paths to the green transformation of Chinese manufacturing enterprises.

**Figure 3:**
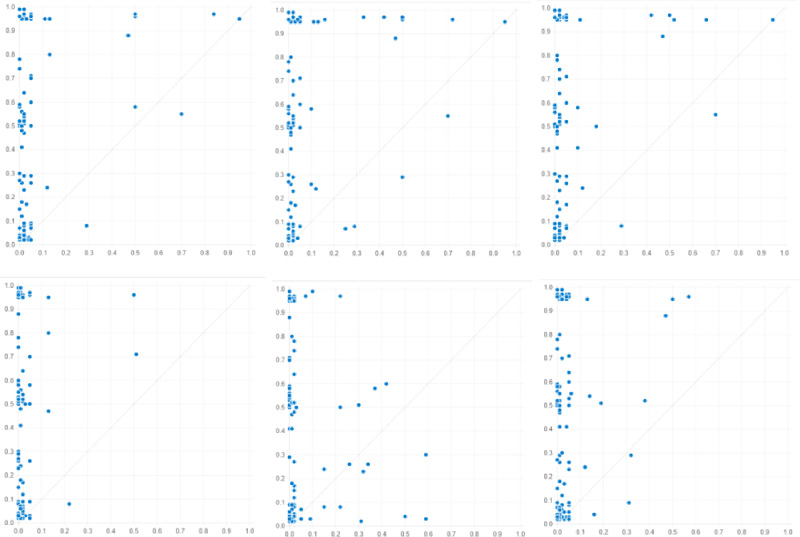
X–Y scatter chart between 6 configurations and the green transformation of manufacturing enterprises.

Based on the aforementioned data calibration and necessity analysis, this article sets the minimum case threshold to 1, the consistency threshold to 0.8, and the PRI consistency threshold to be greater than 0.7^37^. The results of fsQCA software show three solutions, i.e., a complex solution, a minimalist solution, and an intermediate solution, which drive the green transformation of manufacturing enterprises. The intermediate solution considers only simple counterfactual analysis, with a moderate number of configurations and conditions. In QCA research, intermediate solutions are often selected for the interpretation of configuration analysis results. The specific configuration analysis results driving the green transformation of manufacturing enterprises are shown in Table 5. To present the analysis results more clearly, the six paths driving a high level of the green transformation of manufacturing enterprises are visualized through the graph, as shown in Fig. 2. In Fig. 3, this article intuitively depicts the X–Y scatter plot formed by the green transformation of manufacturing enterprises and the six configurations. The case concentrated in the upper left corner is more convincing in reflecting the conclusion that a certain configuration is a sufficient condition for leading to a high level of green transformation of manufacturing enterprises.

**Table 5:** Configuration analysis results.

Configuration	High level of green transformation (GT)					
	HGT1	HGT2	HGT3	HGT4	HGT5	HGT6
GI	●	●	●		⨂	●
DT	●	●		●	⨂	●
RR		⨂	●	●	●	●
SCC	⨂	●	⨂	⨂	●	●
ES	●	⨂	●	●	⨂	●
REGP	⨂	⨂	⨂	⨂	●	●
Consistency	0.9413	0.9155	0.9456	0.9071	0.8685	0.90432
Raw coverage	0.1324	0.0532	0.1297	0.1456	0.1324	0.0910
Unique coverage	0.0261	0.0244	0.0257	0.0432	0.1004	0.0411
Overall solution consistency	0.8944					
Overall solution coverage	0.3812					

●indicates that the core condition exists, ⨂ indicates that the core condition does not exist, ●indicates that the auxiliary condition exists, and ⨂ indicates that the auxiliary condition does not exist. A space indicates that the condition can or cannot exist. The following table is the same.

The conditions that exist in the minimalist solution are referred to as the core conditions of a given configuration, indicating a strong causal relationship with the outcome variables in this study. The remaining conditions that exist in the intermediate solution but do not exist in the simplified solution are called edge conditions, and they have a weak causal relationship with the results. This article adopts the symbolic representation of Ragin and Fiss (i.e., the Fiss configuration diagram)^38^. The consistency of the six high green transformation level configurations in this article is higher than the commonly accepted consistency threshold of 0.8 in existing research. The overall consistency is 0.8944, with a coverage rate of 0.3812, indicating that the six configurations of high green transformation levels have an explanatory power of 89.44% with regard to achieving high levels of green transformation of manufacturing enterprises and can explain 38.12% of the green transformation enterprise cases of high-level manufacturing enterprises. Du and Kim pointed out that in QCA research in the field of management, the coverage of the overall solution based on second-hand data is generally above 0.3. Therefore, the overall coverage of 38.12% in this article meets the requirements^37^.

Configuration 1 (HGT1): Technology-led information synergy-driven green transformation: This path indicates that green technological innovation and digital transformation play a decisive role as core conditions in the process of the green transformation of manufacturing enterprises, with information transparency as an auxiliary condition. In this configuration, the government has formulated relatively loose environmental policies and technical standards, providing greater choices for the green transformation and upgrading of manufacturing enterprises. In a highly complex and uncertain environment, the green transformation of enterprises requires correct decision-making in areas such as green technological innovation, green production, green management, and green supply chains. High information transparency means that manufacturing enterprises' digital transformation can obtain and collect more dynamic information and data on energy conservation, emission reduction, and environmental governance, incorporating more green management decision-making factors into decision-making plans, thereby greatly improving the efficiency of green management decision-making. The improvement in information transparency has created conditions for reducing the concentration of supply chains in manufacturing enterprises. A low supply chain concentration indicates that manufacturing enterprises have diverse online cooperation channels. Resource allocation theory suggests that an increase in online cooperation channels will further allocate various resources and energy, such as human, financial, and material resources, improve resource allocation efficiency, and facilitate the green transformation of enterprises. The high-quality development of the manufacturing industry under the construction of a digital economy and an ecological civilization emphasizes the deep integration and application of digital technology and the control of enterprise carbon emissions. Emerging technologies such as digitalization and green innovation are conducive to eliminating information asymmetry and weakening supply chain concentration, thereby achieving the green transformation of manufacturing enterprises. In summary, this configuration emphasizes the dominance of emerging technologies such as digitalization and green innovation, with information synergy assistance. Therefore, this path is named “technology-led information synergy-driven green transformation”. This configuration explains approximately 13.24% of the cases of a high level of green transformation of manufacturing enterprises. For example, Changan Automobile, which was the first state-owned independent brand automobile manufacturing enterprise in China with both production and sales exceeding one million, has seen its performance increase year by year since its establishment. In 2018, Changan Automobile ranked third among state-owned independent brand automobile manufacturing enterprises, surpassing Beijing Hyundai and GAC Honda. At the same time, Changan Automobile is actively expanding its presence in the fields of intelligence and new energy, collaborating with China Mobile, China Mobile Internet of Things, and Huawei to jointly develop and research LTE-V and 5G vehicle networking. At the same time, it has established a joint venture with Tencent to jointly build an open platform for intelligent vehicle networking, and it has also established a power battery joint venture with BYD to deepen cooperation. Changan Automobile has officially released its “Third Innovation and Entrepreneurship Plan”. The joint research of companies such as Changan Automobile, China Mobile, China Mobile Internet of Things, Huawei, Tencent, and BYD will jointly equip cars with “eyes” and “ears”, create a new Internet of Vehicles ecosystem, and provide opportunities for the green transformation of automotive manufacturing enterprises.

Configuration 2 (HGT2): Technology-led supply chain collaborative-driven green transformation: This path indicates that green technological innovation, digital transformation, and supply chain concentration play a decisive role as core conditions in the process of the green transformation of manufacturing enterprises. In this configuration, environmental governance pressure is relatively low. Under the wave of the digital economy and ecological civilization construction, manufacturing enterprises have placed their strategic focus on promoting green technological innovation through digital transformation. Doing so not only achieves the green transformation and upgrading of manufacturing enterprises but also improves their economic performance. Manufacturing enterprises are undergoing digital transformation under the wave of the digital economy. In terms of green technology innovation, it is beneficial to further develop green and energy-saving technological innovation, reduce energy consumption, achieve enterprise pollution monitoring and warning, and control enterprise wastewater, exhaust gas, and solid waste emissions, thereby promoting enterprise green transformation^39^. In terms of enterprise production and operation, a large amount of data resources are shared, integrated, and effectively utilized, and various links throughout the product lifecycle are optimized and restructured to improve the efficiency of enterprise production and operation as well as resource utilization, to reduce resource waste, and to achieve the green transformation of enterprises. In terms of the enterprise supply chain, digital technology not only enables manufacturing enterprises to break spatial and geographical limitations and expand online cooperation channels^40^ but also facilitates the further rational allocation of various resources and energy, such as human, financial, and material resources, and it improves resource allocation efficiency. Moreover, it increases the number of suppliers in enterprises and limits their bargaining power, which is beneficial for reducing costs and increasing profits. Only then will enterprises have more funds to purchase intelligent devices, promote energy conservation and emission reduction in manufacturing, reduce environmental pollution, and achieve a green transformation. In summary, this configuration emphasizes the dominance of emerging technologies such as digitalization and green innovation, with the supply chain as an auxiliary. Therefore, this path is named “technology-led supply chain collaborative driven green transformation”, which explains approximately 5.32% of the cases of a high level of green transformation of manufacturing enterprises. For example, as a representative industry of traditional manufacturing, steel enterprises have enormous energy consumption and carbon emissions, and green transformation is urgent. In 2018, the Ansteel Group formulated a construction plan for smart Ansteel under the guidance of the development concept of “innovation, coordination, green, openness, and sharing”, as well as Ansteel's high-quality development strategy in the new era. The establishment of smart offices and intelligent manufacturing systems such as ERP (enterprise resource planning) and an MES (manufacturing execution system) enables information interconnection between enterprise management, production manufacturing, factories, and supply chain enterprises. Through the results of big data analysis, production equipment can be transformed or replaced with more intelligent equipment to achieve the goal of saving resources and energy. In addition, Ansteel uses industrial robots to replace virtual positions within the enterprise, significantly improving production efficiency. The digital transformation of production technology has made Ansteel pay more attention to the layout of marketing networks. By building an e-commerce platform to cater to and meet the information needs of suppliers and customers, Ansteel has significantly reduced the procurement time and costs, opened the boundaries of enterprise business activities, optimized resource allocation, improved resource allocation efficiency, and achieved its green transformation.

Configuration 3 (HGT3): Innovation-led organizational collaborative-driven green transformation: This path indicates that green technological innovation, redundant resources, and information transparency play a decisive role as core conditions in the green transformation process of manufacturing enterprises. In this configuration, manufacturing enterprises still face relatively small environmental governance pressures. The development of green innovation strategies encourages manufacturing enterprises to focus their resources on specific activities related to green innovation^41^, which directly affects the green transformation behavior of enterprises. Eiadat et al. pointed out that by implementing environmental innovation strategies, enterprises can promote the effective utilization of manufacturing resources and convert waste into recyclable output through green technological innovation, resulting in an increase in redundant resources^42^. Within an enterprise, redundant resources have a certain buffering effect, enhancing the flexibility of the enterprise, which not only helps to alleviate the cost pressure caused by increased environmental investment in the environmental governance process of manufacturing enterprises but also helps manufacturing enterprises increase their resource investment in green technological innovation. The existence of redundant resources will have a positive impact on the development of green product innovation^12,13^, making it easier for manufacturing enterprises to achieve a green transformation. In addition, by implementing environmental innovation strategies, enterprises can further reduce carbon emissions through green technological innovation. Enterprises can fully disclose carbon information, enabling stakeholders to have a clearer understanding of the achievements and problems of carbon reduction. This is also conducive to obtaining true and reliable information, alleviating information asymmetry, making green management decisions suitable for the development of manufacturing enterprises, and realizing the green transformation of manufacturing enterprises. In summary, this configuration emphasizes the dominance of green technological innovation in enterprises and the synergistic assistance of organizational factors such as redundant resources and information in enterprises. Therefore, this path is named “innovation-led organizational collaborative-driven green transformation”. This configuration explains approximately 12.97% of the cases of a high level of green transformation of manufacturing enterprises. For example, as one of the world's largest cement and building materials enterprises, the Conch Group has continuously increased its investment in research and development innovation, stimulated innovation and creativity, and improved its innovation and creativity in recent years, focusing on the overall goal of “high-end, intelligent, and green” construction. In 2018, the Conch Group took the lead in the cement and building materials industry in “testing” an intelligent cement factory, which increased the enterprise's resource utilization rate by 5%, reduced its energy consumption by 1.2%, and reduced its carbon dioxide emissions by 1.0%. This advantageously promoted the digital, intelligent, and green transformation and upgrading of the Conch Group, as well as its high-quality development.

Configuration 4 (HGT4): Organization-led digital collaborative-driven green transformation: This path indicates that digital transformation, redundant resources, and information transparency play a decisive role as core conditions in the green transformation process of manufacturing enterprises. In this configuration, manufacturing enterprises similar to Configuration 1 face lower environmental governance pressure. Resource-based theory suggests that internal resource factors are more important than external environmental factors for the sustainable development of enterprises. Internal resource integration, knowledge management, and ability enhancement are key conditions for enterprises to obtain sustainable competitive advantages. In the context of intelligent manufacturing, manufacturing enterprises conduct intelligent analysis and formulate and reasonably arrange green production plans and production schedules, fully reducing the energy consumption and resource waste needed in the production process. At this time, redundant resources in the enterprise increase, and the flexibility of the enterprise is enhanced. The theory of resource allocation holds that for enterprises, the key to achieving sustained competitive advantage lies in how to effectively allocate and sustainably utilize their redundant or core resources. The application of digital technology has improved information transparency, which is beneficial for manufacturing enterprises to better reconfigure their redundant resources, improve their resource allocation and utilization efficiency, and thus achieve a green transformation. This path indicates that in the context of a high digital transformation, low supply chain concentration, and low environmental governance pressure, organizational factors such as redundant resources and information transparency play a crucial role in driving manufacturing enterprises to achieve a high level of green transformation. In summary, this configuration emphasizes the dominance of organizational factors such as redundant resources and information within the enterprise and the collaborative assistance of digital transformation. Therefore, this path is named “organizational-led digital collaborative-driven green transformation”. This configuration explains approximately 14.56% of the cases of a high level of green transformation of manufacturing enterprises. For example, as a leading enterprise in the security industry, Hikvision actively transformed in 2018 to break through industry bottlenecks. The main measures consisted of downplaying the concept of security and expanding Hikvision's development into areas such as “the intelligent video IoT” and “video big data”. Its research and development direction has also shifted to focus on breakthroughs in artificial intelligence, cloud computing, and SaaS layers. It has been several years since Hikvision proposed AICLOUD in 2017, and the enterprise has basically achieved edge intelligence. The resource advantages of Hikvision are mainly highlighted in terms of financial resources. In 2018, Hikvision's total operating revenue was 49.8 billion yuan, an increase of 18.86% compared to the previous year. The net profit attributable to shareholders of the listed company was 11.3 billion yuan, an increase of 20.46% compared to the previous year. This provides enormous financial support for Hikvision to carry out green technological innovation and other green transformation measures.

Configuration 5 (HGT5): Organizational-led environmental collaborative-driven green transformation: This path indicates that redundant resources, supply chain concentration, and environmental governance pressure play a decisive role as core conditions in the green transformation process of manufacturing enterprises. In this configuration, manufacturing enterprises face significant environmental governance pressure, and green transformation is more urgent for enterprises than technological transformation such as digitization. The theory of resource allocation holds that for enterprises, the key to achieving sustained competitive advantage lies in how to effectively allocate and sustainably utilize their redundant or core resources. Bowen pointed out that redundant resources can be used to experiment with new environmental innovations or potential green niche markets, enabling the effective implementation of green innovation strategies^43^. Under high environmental governance pressure, the green transformation of manufacturing enterprises is often a passive behavior, requiring a significant increase in environmental investment expenditure. At this time, redundant resources are beneficial for alleviating the cost pressure caused by the increase in environmental investment^12^. In addition, the existence of redundant resources means that enterprises invest more resources and funds in green technological innovation, which is conducive to alleviating the additional cost increase caused by green technological innovation and the pressure of resource and fund shortages in the green transformation process of manufacturing enterprises. The existence of redundant resources in enterprises can also have a positive impact on the development of green product innovation^13^, making it easier for manufacturing enterprises to achieve a green transformation. In terms of the enterprise supply chain, under high environmental governance pressure, the construction and management of a green supply chain are an inevitable trend for the survival and development of contemporary manufacturing enterprises. A green supply chain requires interchain enterprises to implement a series of measures, such as clean production, resource conservation and energy and resource utilization efficiency improvement. This requirement places high demands on suppliers and customers in the supply chain where manufacturing enterprises are located, reducing their dependence on the supply chain. Conversely, bargaining power increases, which is beneficial for reducing costs and increasing profits. At this point, enterprises will have more funds to purchase smart devices, promote energy conservation and emission reduction in manufacturing, reduce environmental pollution, and achieve a green transformation. In summary, this configuration emphasizes that organizational factors such as redundant resources and the supply chain concentration within the enterprise are dominant, and environmental governance pressure is synergistically assisted. Therefore, this path is named “organizational-led environmental collaborative-driven green transformation”. This configuration explains approximately 13.24% of the cases of a high level of green transformation of manufacturing enterprises. For example, in 2018, the sustainable development strategy released by Jizhong Energy included measures in multiple aspects, such as talent cultivation, technological innovation, and social responsibility. Furthermore, Jizhong Energy optimized its energy structure, increased its investment in clean energy, and will further increase the development and utilization of renewable energy to achieve cleaner and more efficient energy management. In terms of the supply chain, Jizhong Energy vigorously promotes the construction of a “smart supply chain” that deeply integrates e-commerce platforms with the internet and the Internet of Things. Enterprises focus on both procurement and sales terminals, not only continuously extending the procurement process but also shifting from retail-based circulation markets to large sales terminals. These measures are conducive to improving energy resource efficiency and accelerating the green transformation and upgrading of enterprises in Jizhong Energy.

Configuration 6 (HGT6): Full-level multielement collaborative-driven green transformation: This path indicates that digital transformation, redundant resources, supply chain concentration, and information transparency play a decisive role in the green transformation process of manufacturing enterprises, with green technological innovation and environmental governance pressure as auxiliary conditions. In this configuration, the popularization of digital information technology has laid the technical foundation for manufacturing enterprises to undergo digital transformation. Under the advocacy of the concept of green development and the acceleration of ecological civilization system reform, manufacturing enterprises utilize technological factors such as green technological innovation and digital transformation to achieve intelligent manufacturing upgrading and clean production, promote energy conservation and emission reduction, and reduce environmental pollution. The development of technology will also lead to changes in organizational factors such as redundant resources, supply chain concentration, and information transparency. Redundant resources are conducive to promoting green product innovation and green technological innovation, green supply chains are conducive to enhancing the bargaining power of enterprises and maintaining supply chain stability, and information symmetry is conducive to enterprises becoming more rational when making green transformation decisions, which in turn will enable manufacturing enterprises to achieve a green transformation. The complexity of redundant resources requires the establishment of appropriate systems for configuration, the diversity of supply chain management requires the establishment of supply chain platforms for management, and the asymmetry of information requires the establishment of comprehensive information platforms for analysis. These organizational factors will force enterprises to undergo digital transformation and conduct green technological innovation. In summary, this configuration emphasizes the six antecedent factors under the TOE theoretical framework to jointly drive the green transformation of manufacturing enterprises. Therefore, this path is named “full-level multifactor collaborative-driven green transformation”, which explains approximately 9.1% of the cases of a high level of green transformation of manufacturing enterprises. For example, according to the 2018 annual report of BOE, the overall shipment volume of BOE display panels remained the world's largest, with a rapid increase in product and yield rates. This outcome was due to the rapid development of the enterprise IoT and the improvement in intelligent manufacturing technology. BOE is further strengthening its capacity building in the transformation and development of the Internet of Things to meet the market demands of different IoT application scenarios and to create a competitive advantage in professional segmented markets, deeply promoting the rapid development of the three major business sectors during the port period, smart IoT, and smart medical industry. In addition, we will accelerate the construction of a sustainable supply chain system, continuously improve the supply chain management process, formulate targeted cooperation strategies, and enhance the competitiveness of the supply chain. These measures of BOE are coordinated and linked, working together to promote BOE's green transformation.

### Robustness test

Based on the practices of most studies, this article tests the robustness of the QCA conclusions above by adjusting the consistency threshold and frequency threshold^37^. First, we adjust the consistency threshold. While keeping the original frequency threshold constant at 1, this article increases the original consistency level from 0.8 to 0.85, which means increasing the consistency threshold by 0.05 before analyzing the case. The results show that after the consistency threshold was increased, the configuration, consistency of solutions, and coverage of solutions generated by manufacturing enterprises in achieving high levels of green transformation do not change. The QCA results are still consistent with the path of achieving high levels of green transformation in the aforementioned manufacturing enterprises. Second, the frequency threshold is adjusted. While maintaining the consistency threshold of 0.8, this article increases the frequency threshold from 1 to 2. The results are shown in Table 6. After adjusting the frequency threshold, the newly generated configuration 1 is a subset of the original QCA result configuration 3, configuration 2 is the original QCA result configuration 5, and configuration 3 is the original QCA result configuration 6. The resulting configuration is a subset of the original configuration.

**Table 6:** Robustness test results.

Configuration	Frequency threshold is 2		
	HGT1	HGT2	HGT3
GI	●	⨂	●
DT		⨂	●
RR	●	●	●
SCC	⨂	●	●
ES	●	⨂	●
REGP	⨂	●	●
Consistency	0.9456	0.8685	0.9043
Raw coverage	0.1297	0.1324	0.0910
Unique coverage	0.0825	0.1057	0.0415
Overall solution consistency	0.8864		
Overall solution coverage	0.2822		

### Discussion

This study uses fsQCA and NCA to explore in depth the complex paths of the green transformation of manufacturing enterprises. This is also consistent with the research findings of “different paths lead to the same goal” proposed by some scholars^44^. But there are also differences. Compared to reference 29, this study applies the TOE theoretical framework to the selection of antecedents for the configuration analysis of the green transformation paths of manufacturing enterprises. Compared to reference 12, this study also selects digital technology as an important antecedent at the technological level. Compared to reference 3, this study chooses the important antecedent of supply chain concentration at the organizational level to explain the role of the supply chain in the green transformation of manufacturing enterprises. Compared to reference 36, this study expands the application field of the indicator of supply chain concentration. For the six transformation paths proposed in this article, compared to reference 28, on the one hand, it also acknowledges the importance of digital technology in the transformation of manufacturing enterprises, and on the other hand, this article is different from its other antecedent conditions in terms of technology, the organization, and the environment and obtains the green transformation paths of manufacturing enterprises.

## Conclusions

In the process of achieving the dual carbon goal in China's entire society, the traditional extensive development model of the manufacturing industry has made it difficult for manufacturing enterprises to achieve a green transformation^2^. The green transformation and upgrading of manufacturing enterprises are a collaborative process of multiple antecedent factors at the three levels of technology, the organization, and the environment. On this basis, improving one aspect alone cannot help manufacturing enterprises achieve a high level of green transformation. Existing research on the paths of the green transformation of manufacturing enterprises focuses on proposing suggestions based only on the linear and nonlinear relationship between a single factor and the green transformation of manufacturing enterprises^3,4,45^. There are different perspectives on the relationship between these single factors and the green transformation of manufacturing enterprises^10^. On the one hand, existing research has not clearly shown what combination of factors can achieve a high level of green transformation in manufacturing enterprises^25^. On the other hand, existing research has not used scientific research methods to propose path choices. To form green and low-carbon core competitiveness for manufacturing enterprises and achieve sustainable development, this study uses fsQCA and NCA to explore in depth the complex paths of the green transformation of manufacturing enterprises. The research conclusions of this article are as follows.

First, green technological innovation, digital transformation, redundant resources, supply chain concentration, information transparency, and environmental governance pressure cannot constitute necessary conditions to drive manufacturing enterprises to achieve a high level of green transformation. Second, there are six paths to achieve a high level of green transformation in manufacturing enterprises, namely, “technology-led information synergy-driven green transformation”, “technology-led supply chain synergy-driven green transformation”, “organization-led organizational synergy-driven green transformation”, “organization-led digital synergy-driven green transformation”, “resource-led environmental collaborative-driven green transformation” and “full-level multifactor collaborative-driven green transformation”. Third, technological factors are crucial for the green transformation of manufacturing enterprises, and the role of technological factors will only be weakened when environmental governance pressure is high. Fourth, organizational factors are crucial for the green transformation of manufacturing enterprises. Fifth, environmental governance pressure is a key external factor in the green transformation of manufacturing enterprises. On the one hand, lower environmental governance pressure provides a greater choice for manufacturing enterprises to promote green transformation behavior. On the other hand, higher governance pressure will force manufacturing enterprises to undergo a green transformation to seek institutional legitimacy.

## Implications for theory and practice

The theoretical significance of this article lies in the following aspects. First, this article introduces the TOE theoretical framework into research on the green transformation of enterprises. This expands the application of the TOE theoretical framework in enterprise strategic management. And also provides a new framework for analyzing the implementation of green transformation strategies for subsequent enterprises. Doing so provides a new framework for analyzing the implementation of green transformation strategies for subsequent enterprises. Second, this research constructs a configuration model of the six antecedent factors that affect manufacturing enterprises' green transformation. This study promotes research on the antecedent conditions and path results of enterprise green transformation and enriches and expands existing research results on enterprise green transformation paths. Third, this article reveals the complex effects of six antecedent factors driving the path of green transformation in manufacturing enterprises..The “black box” of antecedent factors at the three levels of technology, the organization, and the environment is opened up to achieve a high level of green transformation of manufacturing enterprises. This study also responds to the call of scholars such as Wang et al. to “pay attention to the specific process mechanism of enterprises moving towards green”^46^.

The practical significance of this article lies in the following aspects. First, the role of emerging technologies such as digitization and intelligence in the green transformation of manufacturing enterprises is becoming increasingly important. On the one hand, manufacturing enterprises should increase investment in low-carbon technology research and development, and enhance their low-carbon competitiveness through green innovation. On the other hand, we need to develop clean energy and reduce energy consumption per unit product. Second, organizational factors are crucial for the green transformation of manufacturing enterprises. Manufacturing enterprises should use management transformation to promote green transformation. To achieve systematic energy conservation , consumption reduction, and reduce the energy utilization rate of enterprises, manufacturing enterprises should understand their energy consumption and high consumption links through the data system of the control center. Improving the economic and environmental performance of manufacturing enterprises through such measures. Furthermore, from the perspective of supply chain management, building a green supply chain can achieve a full chain green transformation from upstream to downstream. Third, manufacturing enterprises should choose a suitable path for their high-level green transformation based on their inherent antecedent advantages. There are significant differences in the development direction, growth experience, production mode, and life cycle of different enterprises, and the advantages of antecedents are also different. Manufacturing enterprises should use their strengths to compensate for their weaknesses and choose a suitable path for their high-level green transformation based on their advantages in green technological innovation, digital transformation, redundant resources, supply chain concentration, information transparency, and environmental governance pressure, as well as the coupling and coordination relationship between various elements.

## Limitations and future prospects

There are still some limitations to this study. First, this article is limited by time and conditions, and it was not able to explore more factors that affect the green transformation of manufacturing enterprises. It analyzes the configurational path of the green transformation of Chinese manufacturing enterprises from a comprehensive perspective. Second, due to the limited information of the case enterprises, it was not possible to conduct an in-depth analysis of the case enterprises. Third, the antecedent and outcome variables of this study did not consider practical and process factors. These issues all require further research in the future. In particular, we should attach importance to the role of digital technology in future research. Digital technology, characterized by its openness, borderless nature, and strong interactivity in digital scenarios and new features, has become an indispensable part of the green transformation process of manufacturing enterprises^47^. The development of digital technology has provided possibilities for green technological innovation, technological integration, clean production, smart manufacturing, and other aspects of manufacturing enterprises^48^.

## Data Availability

The datasets used and/or analyzed during the current study available from the corresponding author on reasonable request.
